# A point prevalence survey on antibiotic dispensing practices identifies targets for quality improvement of antimicrobial stewardship in community pharmacies in Dar es Salaam, Tanzania

**DOI:** 10.1080/20523211.2026.2691452

**Published:** 2026-07-08

**Authors:** Mary Migiro, Mtebe Majigo, Agricola Joachim, Doreen Kamori, Salim Masoud, Upendo Kibwana, Bjørn Blomberg, Joel Manyahi

**Affiliations:** a Department of Microbiology and Immunology, Muhimbili University of Health and Allied Sciences, Dar es Salaam, Tanzania; b Department of Clinical Science, University of Bergen, Bergen, Norway; c Department of Medicine, National Centre for Tropical Infectious Diseases, Haukeland University Hospital, Bergen, Norway

**Keywords:** Antibiotics, dispensing, community pharmacy, antimicrobial resistance, antimicrobial stewardship

## Abstract

**Background:**

Inappropriate antibiotic use is a major driver of antimicrobial resistance (AMR), especially in low- and middle-income countries (LMICs), where over-the-counter antibiotics are readily available without a prescription. This study assessed antibiotic dispensing practices in community pharmacies in Dar es Salaam, Tanzania.

**Methodology:**

From February to March 2025, a point prevalence survey was conducted in 100 community pharmacies in Dar es Salaam. Researchers approached existing customers, obtained consent, and conducted confidential interviews. Five participants were enrolled per pharmacy, for a total of 500 participants.

**Results:**

Overall, 64% (320/500) of community pharmacy attendees were dispensed antibiotics, of whom 71% (227/320) received them without a prescription. Access-group antibiotics were most frequently dispensed (56%, 201/356), followed by Watch (32%, 115/356) and Reserve groups (3%, 10/356), with metronidazole (15%, 53/356) and azithromycin (12%, 44/356) being the most commonly dispensed antibiotics. Urinary tract infection (UTI) was the leading indication (37%, 119/320), mainly treated with azithromycin (25%, 35/138), ciprofloxacin (12%, 16/138), and nitrofurantoin (7%, 9/138). Among antibiotic recipients, 44% (140/320) received antibiotics based on dispensers’ recommendations. Ceftriaxone was commonly dispensed with prescriptions, while all Reserve antibiotics were dispensed without prescriptions. Participants with technical/vocational education (adjusted prevalence ratio [aPR], 1.38, 95% CI: 1.13–1.68, *p* = 0.001) were more likely to be dispensed antibiotics than those with no formal education or primary and secondary education, and a borderline association was found for college/university education (aPR, 1.22, 95% CI: 0.99 - 1.48, p = 0.05). The presence of an underlying medical condition was an independent protective factor against antibiotic dispensing (aPR, 0.70; 95% CI: 0.49–0.88; *p* = 0.005).

**Conclusion:**

Most clients attending community pharmacies in Dar es Salaam receive antibiotics without prescriptions, often based on dispensers’ recommendations. The widespread use of Watch and Reserve antibiotics without oversight highlights major regulatory gaps and the urgent need for stronger policy enforcement and antimicrobial stewardship.

## Background

Antimicrobial resistance (AMR) is a major global health concern that affects humans, animals, plants, food, and the environment. In the WHO African region, bacterial AMR was the direct cause of 250,000 deaths and associated with a further 700,000 deaths in 2019 (Sartorius et al., 2024). By 2050, it is projected that bacterial AMR will be the direct cause of 1.9 million annual deaths globally and associated with 8.2 million deaths (Naghavi et al., 2024).

One of the factors contributing to the rise of antimicrobial resistance is the inappropriate use of antibiotics, which is widespread across the human, animal, food, and agriculture sectors (Otaigbe & Elikwu, 2023; Rusic et al., 2021; Walsh et al., 2023). Limited access to primary health care, particularly diagnostic services for infectious diseases, leads to increased demand for antibiotics and fuels their inappropriate use in low- and middle-income countries (LMICs) (Kamere et al., 2023; Ndaki et al., 2025). In LMICs, 40–70% of primary health care attendees are dispensed antibiotics, and most of these are inappropriate (Mabilika et al., 2022; Sulis et al., 2020). In Tanzania, studies reveal a high prevalence of antibiotic use in health care facilities, with little use of bacteriological test results (Katyali et al., 2023; Seni et al., 2020). High rates of antibiotic resistance in pathogenic bacteria, consistently being reported in the region, fuel further spread of multidrug-resistant bacteria (Al Masud et al., 2024; Naghavi et al., 2024; Torres et al., 2021).

In LMICs, community pharmacies serve as the primary point of entry for patients seeking healthcare consultations (Mumbi et al., 2024; Thomson et al., 2019). In Tanzania, pharmacies are allowed to dispense prescription-only and over-the-counter medicines, but enforcement of prescription regulations is inconsistent (Mboya et al, 2018). Dispensing antibiotics without a prescription is common practice at these pharmacies (Horumpende et al., 2018b; Mboya et al., 2018; Ndaki et al., 2021). Systematic reviews reported that the prevalence of non-prescription antibiotic use in LMICs ranges from 63% to 79% (Belachew et al., 2022). Previous studies in the Northern and Southern Highlands of Tanzania have reported high rates of nonprescription dispensing (88%–92%) among community pharmacies using simulated clients (mystery clients) (Horumpende et al., 2018b; Mboya et al., 2018; Ndaki et al., 2021).

The second strategic objective of Tanzania's National Action Plan for AMR (NAP) addresses the need to strengthen knowledge and evidence of AMR and antibiotic use through surveillance (United Republic of Tanzania: Second national action plan on antimicrobial resistance 2023–2028, 2022). Surveillance networks for antimicrobial resistance have been rolled out across human, animal, and aquaculture settings, and the data generated are used to update guidelines and inform policy changes (United Republic of Tanzania. National Antimicrobial Resistance Framework, 2018). However, to our knowledge, only a few surveys have documented antimicrobial use practices in community pharmacies involving real-world patients. Community pharmacies are often the initial point of contact for patients seeking health care, and understanding their practices regarding antibiotic use is critical for addressing the drivers of antimicrobial resistance. In Tanzania, pharmacies function as a hybrid between formal healthcare and community-based service, bridging gaps caused by limited hospital capacity, long distances to clinics, and affordability barriers (Horumpende et al., 2018b; Mboya et al., 2018; Ndaki et al., 2021). Understanding this context is crucial for interpreting patterns of medicine use. This study aims to investigate patterns of antibiotic use in community pharmacies using real-world patients seeking medical attention at these facilities.

## Material and methods

### Study design, setting, and population

This was a point-prevalence survey conducted in community pharmacies in Dar es Salaam from February 2025 to March 2025. Participants included attendees who visited community pharmacies on the day of the point prevalence survey. Children below 18 years were excluded as it was not deemed feasible to obtain parental consent and child assent in this study setting. Dar es Salaam is the biggest city in Tanzania; it comprises five municipalities: Ilala, Kinondoni, Ubungo, Temeke, and Kigamboni. It has a population of 8.6 million and serves as the country’s main economic hub. The city has a growing healthcare sector, with numerous polyclinics and 1,597 registered community pharmacies that serve its residents’ healthcare needs.

### Sampling method

According to the Pharmacy Council registration database, 1,141 (71%) of the 1,597 registered community pharmacies in Dar es Salaam are currently operating. These are distributed across five municipalities as follows: Kinondoni (385), Ilala (310), Ubungo (206), Temeke (157), and Kigamboni (83). A sample of 100 community pharmacies was selected based on the number of pharmacies that could feasibly be visited within the available time. To ensure even representation across municipalities, the principle of probability proportional to size (PPS) was applied. The sampling fraction was calculated by dividing the sample size of 100 by the total number of registered and operating pharmacies (1,141), resulting in a proportion of approximately 8.8%. This proportion was then applied to each municipality’s total number of pharmacies, yielding the following distribution of sampled pharmacies: 34 from Kinondoni, 27 from Ilala, 18 from Ubungo, 14 from Temeke, and 7 from Kigamboni. Within each municipality, the required number of pharmacies was selected using systematic random sampling. First, a complete list of registered pharmacies in each district was obtained. The lists were then randomised by shuffling to minimise order bias. From each selected pharmacy, five (5) clients were selected consecutively by convenience sampling. That yields a total sample size of 500 clients across all selected community pharmacies.

### Data collection

Data were collected using a validated structured questionnaire adapted from Nabeel et al. (2024) to capture relevant client information. The questionnaire covered sociodemographic characteristics (age, sex, education) and medical/clinical details, including the antibiotic dispensed, prescription status, and source of recommendation (self, pharmacist, or health professional). It was digitised using Kobo Toolbox and pilot-tested with 10 participants to assess clarity, flow, and sequencing, which led to revisions before the main study. Pharmacies were informed in advance about the study, but data collection began two to three weeks later to minimise behaviour change. Informed consent and face-to-face interviews were obtained directly from clients by trained assistant researchers, who positioned themselves discreetly outside pharmacies and interviewed consenting clients privately as they exited.

### Statistical analysis

Data were collected using Kobo Toolbox. Analysis was performed using Statistical Package for Social Sciences (SPSS) version 29.0 *(IBM Corp. IBM SPSS Statistics for Windows, Version 29.0. Armonk, NY: IBM Corp; 2022)*. Frequencies and proportions were used to summarise categorical variables; medians and interquartile ranges were used to summarise continuous variables; and chi-square tests were used to assess associations between categorical variables and antibiotic dispensing practices. A modified Poisson regression model was used to identify factors associated with antibiotic dispensing practices, and effect estimates are presented as crude and adjusted prevalence ratios (cPR and aPR, respectively) with 95% confidence intervals (95% CI). Variables with a *p*-value less than 0.20 in the bivariate analysis were included in the multivariable model, and a *p*-value of less than 0.05 was considered statistically significant. Potential confounders, such as age, sex, and underlying medical conditions, were controlled for in the multivariable analysis.

### Ethical consideration

Ethical approval was obtained from the Muhimbili University of Health and Allied Sciences (MUHAS) Senate Research and Publication Committee with a reference number DA.282/298/01.C/2546. Permission to conduct the study was also obtained from the Pharmacy Council of Tanzania. Written informed consent was obtained from participants.

## Results

### Baseline demographics and medical characteristics of the study participants

In this study, a total of 500 participants were enrolled from community pharmacies in Dar es Salaam. Participants’ median age was 30 years (interquartile range of 25–37), and 40% (201/500) were aged 25–34 years (Table 1).

**Table 1.** Demographics and medical characteristics of the study participants.

Variables	Frequency N = 500	Percentage (%)
**Age group (year)**		
18–24	121	24
25–34	201	40
35–44	110	22
35–54	44	9
>54	24	5
**Median age in years (IQR)**	30 (25–37)	
Gender		
Male	243	49
Female	257	51
**Marital status**		
Single	246	49
Married	254	51
**Residence (Districts)**		
Ilala	135	27
Kigamboni	35	7
Kinondoni	170	34
Temeke	70	14
Ubungo	90	18
**Education level**		
No formal education	19	4
Primary	48	10
Secondary	132	26
Technical/Vocational	70	14
College	231	46
**Occupation**		
Students	101	20
Self-employed	261	52
Private-company employee	95	19
Government employee	43	9
**Health insurance**		
Yes	142	28
No	358	72
**Underlying conditions**		
Yes	44	9
No	456	91
Current on medications*		
Yes	78	16
No	422	84

IQR (Interquartile Range) * Long/short term medications (antibiotics, antifungal, antimalarials and chronic disease medications).

Of all participants, 51% (257/500) were female, 34% (170/500) resided in Kinondoni municipality, 51% (254/500) were married, and 46% (231/500) had a college or university education. Among all, 52% (261/500) were self-employed, 84% (422/500) were not on any medications, and 9% (44/500) had underlying medical conditions, including hypertension 59% (26/44), diabetes 18% (8/44), chronic kidney disease 9% (4/44), and heart disease 9% (4/44), while 5% (2/44) had other underlying conditions. Overall, 72% (358/500) had no health insurance coverage. Among those with health insurance coverage, 96% (136/142) were covered by the government's National Health Insurance Fund (NHIF) (Table 1).

### Prevalence of antibiotics dispensed at the community pharmacies in Dar es Salaam

Sixty-four percent (320/500, 95% confidence interval (CI) 59.6-68.2) of the community pharmacy attendees were dispensed antibiotics, 7% (33/500) were dispensed antifungals, 27% (136/500) were dispensed analgesics, 9% (46/500) were dispensed anti-allergy medications, 4% (22/500) were dispensed supplements, and 3% (17/500) were dispensed other medications. Antibiotic dispensing was more frequent in the youngest age groups, ranging from 64% (77/121) in those aged 18–24 years to 4% (1/24) in those older than 54 years. There was no significant difference in antibiotic dispensing by sex (67%, 172/257 in females, 61%, 148/243 in males, *p* = 0.230). A significant variation in antibiotic dispensing was observed across districts (*p* < 0.001), with the highest prevalence in Kigamboni, 83% (29/35, Table 2). There was a significant variation in antibiotic dispensing by education status, with the highest prevalence among participants with technical/vocational education (79%, 55/70; *p* = 0.001). Antibiotic dispensing also varied by occupation, with the highest prevalence noted among government employees (81%, 35/43, *p* < 0.001). Notably, lower antibiotic dispensing was observed among participants with underlying conditions (30%, 13/44), compared to those without underlying conditions (67%, 307/456, *p* < 0.001, Table 2).

**Table 2.** Factors influencing antibiotic dispensing practice in the community pharmacies in Dar es Salaam.

Variable	Total participants (N)	Participants dispensed antibiotics (n)	Prevalence (%)	Univariate analysis			Multivariate analysis		
				cPR	95% CI	*p*-value	aPR	95% CI	*p*-value
Age (Years)	500	320	64	0.99	0.98–1.00	0.05	1.00	0.99–1.01	0.91
**Gender**									
Male	257	172	67	1.10	0.96–1.25	0.163	1.07	0.91–1.25	0.14
Female	243	148	61	Ref					
**Education status**									
No formal education	19	12	63	1.29	0.84–1.79	0.29	1.16	0.79–1.71	0.43
Primary	48	28	58	1.13	0.85–1.51	0.40	1.18	0.89–1.57	0.23
Technical/vocational	70	55	78	1.53	1.24–1.87	**<0**.**001**	1.38	1.13–1.68	**0**.**001**
College/University	231	157	68	1.32	1.09–1.59	**0**.**004**	1.22	0.99–1.48	0.05
Secondary	132	68	52	Ref					
**Districts**									
Ilala	135	106	79	1.61	1.28–2.02	**<0**.**001**	1.43	1.15–1.78	**0**.**001**
Kigamboni	35	29	83	1.69	1.31–2.19	**<0**.**001**	1.42	1.10–1.83	**0**.**006**
Kinondoni	170	96	56	1.16	0.90–1.48	0.26	1.05	0.83–1.33	0.678
Temeke	70	45	64	1.32	1.00–1.73	0.05	1.19	0.92–1.55	0.180
Ubungo	90	44	49	Ref					
**Occupation**									
Students	101	75	74	0.91	0.76–1.09	0.33	0.10	0.86–1.42	0.447
Self employed	261	162	62	0.76	0.64–0.91	**0**.**002**	0.86	0.71–1.04	0.112
Private company	95	48	51	0.62	0.49–0.79	**<0**.**001**	0.75	0.59–0.93	**0**.**011**
Government employee	43	35	81	Ref					
**Insurance status**									
Insured	142	99	70	1.13	0.99–1.29	0.079	1.03	0.87–1.23	0.704
Uninsured	358	221	62	Ref					
**Underlying condition**									
Yes	44	13	30	0.44	0.28–0.70	**< 0.001**	0.70	0.49–0.88	**0**.**005**
No	456	307	67	Ref					

cPR: crude Prevalence Ratio, aPR: adjusted Prevalence Ratio, Ref: Reference category.

### Patterns and indications of antibiotics dispensing practices at community pharmacies

Among all who were dispensed antibiotics, 15% (48/320) were already on antibiotics and came for a refill of the same antibiotics, and 85% (272/320) were dispensed antibiotics as the first dose. Oral antibiotic administration was more common (84%, 268/320) than injections (11%, 35/320) and mixed prescriptions of both parenteral and oral antibiotics (1%, 4/320). The majority, 90% (287/320), were dispensed a single antibiotic, 68% (217/320) purchased the full dosage, and 71% (227/320) were dispensed without a prescription. The most common indication for antibiotic dispensing was urinary tract infection (UTI), accounting for 37% (119/320), and most of the participants, 44% (140/320), were dispensed antibiotics on the recommendation of the dispenser (Table 3).

**Table 3.** Patterns, indications, and reasons of antibiotic dispensing practices at community pharmacies.

Variable	Frequency (n)	Percentage (%)
**Refill of antibiotics**		
Yes	48	15
No	272	85
**Route of administration of antibiotic dispensed**		
Oral	268	84
Injections/parenteral	35	11
Topical	13	4
Oral + injection	4	1
**Dosage of antibiotics purchased**		
Full dosage	217	68
Incomplete dosage	103	32
**Antibiotic dispensed with prescription**		
Yes	97	30
No	223	70
**Indication for dispensing antibiotics**		
Fever	35	11
GTI	71	22
UTI	119	37
RTI	82	26
Pain management^a^	21	7
Other	59	18
**Who recommended antibiotics**		
Dispenser	140	44
The client herself / himself	66	21
Relative	17	5
Visited hospital	97	30
**Number of antibiotics dispensed**		
One	287	90
More than one	30	9

GTI: gastrointestinal tract infection, UTI: urinary tract infection, RTI: respiratory tract infection.

^a^ Antibiotics used for pain management were mainly for ear infections (n = 9), ostealgia (n = 7) and odontogenic pain (n = 5).

### Frequency of antibiotics dispensed by the community pharmacies

**Figure 1.**
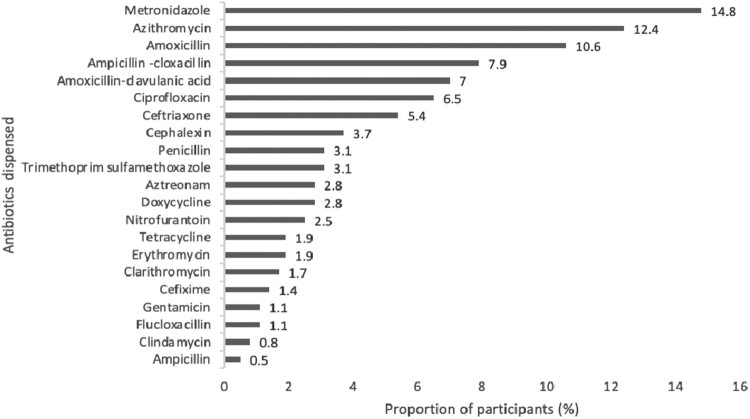
Proportion of participants who were dispensed antibiotics.

Of 320 participants dispensed antibiotics, a total of 21 different antibiotics were dispensed. The majority, 90% (287/320) received one antibiotic, 9% (30/320) a combination of two, and 1% (3/320) a combination of three, bringing an overall frequency of 356 antibiotics prescribed in total. The most frequently dispensed antibiotics were metronidazole 15% (53/356), azithromycin 12% (44/356), amoxicillin 11% (38/356), and ampicillin-cloxacillin 8% (28/356) (Figure 1).

### Antibiotics dispensed per specific indications

The most commonly dispensed antibiotics for presumed urinary tract infection were azithromycin (25%, 35/138), ciprofloxacin (12%, 16/138), nitrofurantoin (7%, 9/138), and ceftriaxone (7%, 9/138). For presumed diagnoses of respiratory tract infections, the antibiotics dispensed were primarily ampicillin-cloxacillin (26%, 23/89) and amoxicillin (16%, 14/89). Metronidazole (51%, 36/71) and amoxicillin-clavulanic acid (13%, 9/71), were most commonly dispensed for presumed gastrointestinal tract infections. For other indications such as fever and pain management, amoxicillin was commonly dispensed in 26% (10/38) and 22% (5/23) of cases, respectively (Table 4).

**Table 4.** Common antibiotics dispensed per specific indications/reasons.

Indication for antibiotics	Antibiotics	Frequency (n)^a^	Percentage (%)
UTI (N = 138)	Azithromycin	35	25
	Ciprofloxacin	16	12
	Nitrofurantoin	9	7
	Ceftriaxone	9	7
	Aztreonam	9	7
RTI (N = 89)	Ampicillin-cloxacillin	23	26
	Amoxicillin	14	16
	Cephalexin	8	9
	Azithromycin	7	8
GTI (N = 71)	Metronidazole	36	51
	Amoxicillin-clavulanate	9	13
	Amoxicillin	8	11
	Ciprofloxacin	4	6
Pain management (N = 23)	Amoxicillin	5	22
	Tetracycline	2	9
	Ciprofloxacin	2	9
	Azithromycin	2	9
Fever (N = 38)	Amoxicillin	10	26
	Azithromycin	8	21
	Ceftriaxone	4	11

^a^ Numbers of antibiotics dispensed in the brackets exceeded the total number of participants with specific indication because some received more than one antibiotic. UTI: urinary tract infection, RTI: respiratory tract infection, GTI: gastrointestinal tract infection.

With regard to the WHO AWaRe classification of antibiotics, most antibiotics dispensed were in the Access category, 56% (201/356); 32.3% (115/356) were in Watch categories, and 3% (10/356) were in the Reserve category. Antibiotics from the Watch group were most frequently recommended by the dispenser.

### Prescription status of antibiotics dispensed in the community pharmacies

Two thirds of antibiotics (67%, 238/356) were dispensed without prescriptions. The antibiotics that were most commonly dispensed without prescription were aztreonam (100%, 10/10), amoxicillin (82%, 31/38), ampicillin-cloxacillin (79%, 22/28), azithromycin (77%, 34/44), and metronidazole (76%, 40/53) Conversely, ciprofloxacin (77%, 10/13) and ceftriaxone (74%, 14/19) were the antibiotics most frequently dispensed with valid prescription.

Forty percent (46/115) of watch antibiotics (40%, 46/115) were dispensed with valid prescriptions compared to 32% of access antibiotics (64/201), but the difference was not statistically significant (*p* = value 0.180). All reserve categories of antibiotics dispensed in this study were without prescriptions.

### Factors influencing antibiotic dispensing practice in the community pharmacies in Dar es Salaam

In the univariate analysis, participants with technical/vocational education (cPR 1.32, 95% CI: 1.09–1.59, *p* < 0.001) and those with college/university education (cPR 1.53, 95% CI: 1.24–1.870, *p* < 0.004) were more likely to be dispensed antibiotics compared to those with secondary education. After adjusting for potential confounders, participants with technical/vocational education (aPR 1.38, 95% CI: 1.13–1.68, *p* = 0.001) remained more likely to be dispensed antibiotics, and a borderline association was remained for those with college/university education (aPR 1.22, 95% CI: 0.99 - 1.48, p = 0.05).

Participants from Ilala (cPR 1.61, 95% CI: 1.28–2.02, *p* < 0.001) and Kigamboni (cPR 1.69, 95% CI: 1.31–2.19, *p* < 0.001) were more likely to be dispensed antibiotics compared to those from other districts. After controlling for potential confounders, participants from Ilala (aPR 1.43, 95% CI: 1.15–1.78, *p* = 0.001) and Kigamboni (aPR 1.42, 95% CI: 1.10–1.83, *p* = 0.006) remained more likely to be dispensed antibiotics.

In terms of occupation, both self-employed participants (cPR 0.76, 95% CI: 0.64–0.91, *p* = 0.002) and those employed in private companies (cPR 0.62, 95% CI: 0.49–0.79, *p* < 0.001) were less likely to be dispensed antibiotics compared to other groups of occupation. After adjusting for potential confounders, only employment in private companies remained less likely to be dispensed antibiotics (aPR 0.75, 95% CI: 0.59–0.93, *p* = 0.011).

Conversely, participants having an underlying condition were less likely to be dispensed antibiotics (cPR 0.44, 95% CI: 0.28–0.70, *p* < 0.001). On multivariable analysis, the presence of an underlying condition remained independently associated with reduced likelihood to be dispensed an antibiotic (aPR 0.70, 95% CI: 0.49–0.88, *p* = 0.005, Table 2).

## Discussion

This point prevalence survey involving 500 participants visiting 100 community pharmacies in Dar es Salaam revealed a high prevalence of antibiotic dispensing, with two-thirds of all participants receiving antibiotics without prescriptions. This is in line with pooled prevalence of community pharmacy antibiotic dispensing from recent systematic reviews from LMICs (Li et al., 2023; Torres et al., 2021), and the findings reflects a widespread and irrational practice that continues to fuel antimicrobial resistance (AMR) in the region. Previous studies from Tanzania (Horumpende et al., 2018b; Mboya et al., 2018) have shown even higher prevalences of non-prescription antibiotic dispensing (80-90%). The difference may be due to differences in study design. The previous Tanzanian studies, which reported higher rates of antibiotic use, relied on simulated patients (mystery clients) with specific syndromes to assess dispensing practices in community pharmacies (Horumpende et al., 2018b; Mboya et al., 2018; Ndaki et al., 2021) . Using simulated participants may increase the demand for antibiotics (Horumpende et al., 2018b; Mboya et al., 2018; Sono et al., 2023). A strength of our study is that we interviewed actual participants who visited these pharmacies on their own, which may increase the reliability of the results. The fact that study personnel informed participating pharmacies about the study upfront may have contributed to more stringent dispensing in the study period, but we believe the effect of this information to be minimal since it was given several weeks in advance. Additionally, public campaigns like ‘Holela Holela Itakukosti’ (Recklessness is costly), launched in 2024 and focused on the appropriate use of antimicrobials (Lwakatare, n.d.), have increased awareness of the drivers of AMR. Our study confirms that community pharmacies remain a key access point for non-prescription antibiotics, emphasising the need for enhanced regulation and targeted antimicrobial stewardship training targeting community pharmacies. These results reinforce the ongoing concern that without sustained enforcement and public education, community pharmacies will continue to fuel inappropriate antibiotic use and foster antimicrobial resistance (Belachew et al., 2022).

Dispensers were the most common source of antibiotic recommendations in this survey. This aligns with previous reports from Tanzania and other LMICs, which noted the influence of dispensers in driving non-prescription sales of antibiotics (Auta et al., 2019; Belachew et al., 2021; Horumpende et al., 2018b; Kagashe et al., 2011; Li et al., 2023). This practice is largely influenced by their role as primary healthcare providers and business operators. Although community pharmacies are not considered a part of the primary healthcare system in Tanzania (Tanzania - Strengthening Primary Health Care for Results Program Project (English), n.d.), they are often the most convenient and easily accessible first point for patients seeking healthcare advice. Similarly, dispensers with limited or no formal pharmaceutical training routinely recommend antibiotics, driven more by commercial interests than by clinical appropriateness (Nabeel et al., 2024; Sarwar et al., 2018). These findings suggest that the level of knowledge and professional training among dispensers significantly influences their antibiotic recommendations, but business motivations often override rational prescribing practices (Acharya et al., 2021; Belachew et al., 2021; Horumpende et al., 2018b). Our findings reaffirm that community pharmacies are not merely passive suppliers but active participants in the provision of inappropriate antibiotics. Addressing this issue requires targeted interventions that combine education, regulation, and a shift in the profit-driven dispensing culture practice in many community pharmacies. Future interventions should focus on educational and antimicrobial stewardship programs targeting community pharmacy workers. These programs could provide updated knowledge on rational antibiotic use, guidelines for appropriate dispensing, including adherence to the standard treatment guideline and WHO AWaRe guideline; and strategies to educate customers about responsible antibiotic consumption.

Metronidazole, azithromycin, and amoxicillin were the most dispensed antibiotics in this community settings, aligning with other studies (Al Masud et al., 2024; Ndaki et al., 2021; Rabbani et al., 2023). Ampicillin-cloxacillin and amoxicillin remain the top antibiotics for symptoms suggestive of respiratory tract infections (Auta et al., 2019; Ndaki et al., 2021), while azithromycin, surprisingly, is the most commonly prescribed drug for urinary tract infections (Al Masud et al., 2024; Aziz et al., 2021; Kagashe et al., 2011). These dispensing patterns were not in line with Tanzania’s standard treatment guidelines (Standard Treatment Guidelines and National essential medicines list for Tanzania Mainland, n.d.). Particularly, azithromycin is not among the recommended antibiotics for UTI, although it is used for sexually transmitted diseases, which can feature dysuria, a common symptom with UTI, and are diagnoses which participants may be less willing to reveal to study staff. Consequently, some cases classified as UTI in this study may have actually represented misdiagnosed or unreported sexually transmitted diseases, which could explain the high observed use of azithromycin. Additionally, both patients and unskilled pharmacy staff may lack sufficient medical knowledge to link symptoms to the correct diagnoses. As azithromycin is a broad-spectrum antibiotic, effective against a range of infections including respiratory infections and typhoid fever, it may be preferred by dispensers for patients with unclear diagnoses who lack prescriptions. These findings show that dispensers do not follow guidelines when deciding which antibiotics to dispense, further contributing to irrational antibiotic use. Studies have shown that most community pharmacy dispensers rarely consult or adhere to treatment guidelines in their daily practice, relying instead on informal knowledge or prior experience (Miller & Goodman, 2016; Ndaki et al., 2021). This highlights the urgent need for training dispensers on standard treatment guidelines and the national essential medicines list to ensure rational dispensing practices.

We found that 56% of antibiotics dispensed in community pharmacies were in the WHO Access group, which is well below the 70% target set by the United Nations General Assembly (UNGA) Political Declaration for 2030 (UN General Assembly-High level meeting on AR, 2024). National antibiotic consumption data and other data from Tanzania also show that, in 2022, 50-54% of antibiotics used were in the WHO access group of antibiotics (Global Antimicrobial Resistance and Use Surveillance System (GLASS) report antibiotic use data for 2022, 2022; Seni et al., 2020; Zimbwe et al., 2024), which is consistent with our findings. Based on our findings, the lower proportion of consumption of Access group antibiotics indicates a greater share and widespread reliance on Watch and Reserve antibiotics, posing risks for the development of antimicrobial resistance. The use of Reserve antibiotics without a prescription in this study raises concern, as these should be reserved for confirmed multi-drug-resistant infections and used as last-resort antibiotics, not for presumed infections based on self-diagnosis and advice from pharmacy workers without medical qualifications. Our findings underscore the existing gaps in knowledge regarding the WHO AWaRe classification of antibiotics (World Health Organization, 2023), and its recommendations in our community pharmacies. Therefore, our AMR national action plan should emphasise strengthening antimicrobial stewardship to improve dispensing practices and integrating WHO AWaRe stewardship principles into community pharmacy practices.

Participants with technical/vocational and college/university education were more likely to be dispensed antibiotics, often without prescriptions, influenced by dispenser recommendations and their own assumptions. Many with technical/vocational education lacked health insurance and frequently self-medicated to avoid out-of-pocket hospital costs, while about half of the college/university group, despite having insurance, sought antibiotics over the counter, likely to avoid hospital queues or due to past experiences. These results suggest that higher education does not necessarily promote rational antibiotic use (Acharya et al., 2021), as seen in other studies where those with more education are more prone to self-medication (Gabriel & Balakrishna, 2021; Horumpende et al., 2018a). In Tanzania, participants with higher education were 1.6 times more likely to self-medicate despite better knowledge (Shitindi et al., 2024). Similar trends were noted internationally (Acharya et al., 2021; Belachew et al., 2021; Ekambi et al., 2019), possibly driven by client pressure on dispensers (Acharya et al., 2021) , though one study found higher self-medication among those with no formal education (Bogale et al., 2019). Addressing this requires education efforts, as emphasised in Tanzania’s National Action Plan on AMR (2023-2028) (The national Action Plan on Antimicrobial Resistance 2023-2028, n.d.), and stricter pharmacy-level enforcement of prescription-only policies.

In contrast, having an underlying condition appeared to be a protective factor against being dispensed antibiotics without a prescription in this study, particularly among older adults (>54 years) with chronic illnesses. Their lower rates of antibiotic use likely reflect the higher likelihood that they attend the pharmacy to collect medicines for their underlying disease than for intercurrent infections, as well as possibly a greater engagement with formal healthcare, regular follow-ups, and higher health care awareness. Similar patterns have been reported in LMICs (Bogale et al., 2019; Widayati et al., 2011).

Strengths of the study were the relatively large sample size, the study design with randomised selection of study sites according to the principle of probability proportional to size, as well as the inclusion of real patients instead of simulated patients. One limitation of the study was that all included community pharmacies were within the city of Dar es Salaam only. Secondly, participants were recruited from community pharmacies using a convenience sampling approach, which may introduce selection bias related to healthcare-seeking behaviour and timing of visits. Individuals who attend pharmacies at specific times or who are more likely to seek care may have been overrepresented. Finally, a fixed number of clients per pharmacy was included, which may have resulted in unequal selection probabilities across pharmacies. These findings should be interpreted as representative of community pharmacy attendees rather than the general population. Recruitment of participants from diverse sociodemographic backgrounds improves variability, but does not guarantee full representativeness. Therefore, the findings may not be generalisable to other regions of Tanzania with different healthcare-seeking behaviours and regulatory enforcement levels. Despite these limitations, the study provides valuable insights into antibiotic dispensing practices in community pharmacy settings and highlights critical areas for stewardship interventions.

## Conclusion

Our study shows that non-prescription antibiotic dispensing remains common in community pharmacies. Although most dispensed antibiotics were in the WHO Access category, use remained below the 70% target, and dispensing practices appeared to vary according to clients’ educational attainment and underlying health conditions, underscoring the interplay between patient-related factors and pharmacy dispensing behaviour. These findings highlight the dual challenge of regulating pharmacy practices and addressing client demand through targeted education and public health interventions.

## Ethics approval and consent to participate

Obtained from the Muhimbili University of Health and Allied Sciences (MUHAS) Senate Research and Publication Committee with a reference number DA.282/298/01.C/2546. Written informed consent was obtained from participants.

## Data Availability

The dataset analysed during the current study is available from the corresponding author on reasonable request.
